# Understanding community health worker incentive preferences in Uganda using a discrete choice experiment

**DOI:** 10.7189/jogh.11.07005

**Published:** 2021-03-10

**Authors:** Smisha Agarwal, Timothy Abuya, Richard Kintu, Daniel Mwanga, Melvin Obadha, Shivani Pandya, Charlotte E Warren

**Affiliations:** 1 Department of International Health, the Johns Hopkins University Bloomberg School of Public Health, Baltimore, Maryland, USA; 2 Population Council, Nairobi, Kenya; 3 Formally Pathfinder International, Kampala, Uganda; 4 Health Economics Research Centre, Nuffield Department of Population Health, University of Oxford, Oxford, United Kingdom; 5 University College, Oxford, UK; 6 Population Council, Washington, D.C., USA

## Abstract

**Background:**

Community health workers (CHWs) play a critical role in supporting health systems, and in improving the availability and accessibility to health care. However, CHW programs globally continue to face challenges with poor performance and high levels of CHW attrition. CHW programs are often underfunded and poorly planned, which can lead to loss of motivation by CHWs. The study aims to determine preferences of CHWs for job incentives with the goal of furthering their motivation and success.

**Methods:**

Relevant incentive attributes were identified through focus group discussions and in-depth interviews with CHWs, non-governmental organization CHWs, CHW supervisors, and policy-level stakeholders. Based on seven attributes (eg, training, workload, stipend) we developed a discrete choice experiment (DCE) that was administered to 399 CHWs across eight districts in Uganda. We used conditional and mixed multinomial logit models to estimate the utility of each job attribute. We calculated the marginal willingness to accept as the trade-off the CHWs were willing to make for a change in salary.

**Results:**

CHWs preferred higher salaries, though salary was not the most important attribute. There was a preference for reliable transportation, such as a bicycle (β = 1.86, 95% CI = 1.06, 2.67), motorcycle (β = 1.81, 95% CI = 1.27, 2.34) or transport allowance (β = 1.37, 95% CI = 0.65, 2.10) to no transport. Formal identification including identity badges (β = 1.61, 95% CI = 0.72, 2.49), branded uniforms (β = 1.04, 95% CI = 0.45, 1.63) and protective branded gear (β = 0.76, 95% CI = 0.32, 1.21) were preferred compared to no identification. CHWs also preferred more regular refresher trainings, the use of mobile phones as job-aids and a lesser workload. The relative importance estimates suggested that transport was the most important attribute, followed by identification, refresher training, salary, workload, recognition, and availability of tools. CHWs were willing to accept a decrease in salary of USH 31 240 (US$8.5) for identity badges, and a decrease of USH85 300 (US$23) for branded uniforms to no identification.

**Conclusions:**

This study utilized CHW and policymaker perspectives to identify realistic and pragmatic incentives to improve CHW working conditions, which is instrumental in improving their retention. Non-monetary incentives (eg, identification, transportation) are crucial motivators for CHWs and should be considered as part of the compensation package to facilitate improved performance of CHW programs.

Community health workers (CHWs) play an important role in addressing critical inequities in health care access and support the linkage of communities to health care services. They are often the first point of contact communities have with health systems [1]. In 2001, Uganda introduced their community health workforce, known as Village Health Teams (VHTs). VHTs are responsible for health promotion, health education, community mobilization for health service utilization, community case management and follow up, and the distribution of health commodities to support advancement of maternal and child health, as well as, more broadly, primary health care [2]. As of 2015, over 179 000 VHTs have been trained since the program’s inception, and operate in all 112 districts in Uganda [2,3]. The VHT program is supported by Uganda’s Ministry of Health (MoH), as well as a number of non-governmental development organizations (eg, United Nations agencies, Pathfinder International, AMREF) financially and logistically [2]. Non-governmental organizations (NGOs) also have their own CHWs, often known as community health promoters (CHPs), that provide support, education, and services to the community.

Uganda’s VHT program has shown considerable successes, demonstrated by improvements in access to health services and health outcomes at the community level [4,5]. However, despite VHTs increasing the accessibility and availability of health care in Uganda, the program itself is facing high levels of attrition – similar to many CHW programs globally [2,4]. It is estimated that over 30% of VHTs have dropped out since inception and several continue to only be active during special campaigns [4]. Although CHWs play an integral role in strengthening primary health care (PHC), CHW programs are often underfunded and poorly supported [5]. Heavy workloads (often exacerbated by those high attrition rates), poor supervisory and logistical support, lack of training and recognition, and inadequate compensation and incentive structures all serve to demotivate CHWs and detrimentally affect retention [1,4-7]. In Uganda, VHTs are volunteers; they are not paid for their work, often having other jobs alongside their role as a VHT. VHTs may be given non-monetary incentives to motivate their work, with the hope that it will help improve their morale, performance, and retention. A recent assessment of the VHT program suggests that VHTs receive monetary incentives, through allowances for transportation and meals – but this was neither frequent nor consistent [2]. VHTs also receive non-monetary incentives in the form of branded clothing, protective gear (eg, raincoats, gumboots), transportation (eg, bicycle), but this too was neither frequent nor consistent [2]. Given the issues outlined above, it was recommended that the current-standing VHT strategy be reviewed and improved to better meet the needs of VHTs [4].

In response to the recommendations outlined in the *National Village Health Teams (VHT) Assessment in Uganda* report, Uganda’s MoH introduced the plan for a policy roll-out that would create a new CHW cadre known as “Community Health Extension Workers” (CHEWs) [8]. CHEWs, in contrast to VHTs, would be paid and work full-time but would have to meet stricter selection criteria [8]. This announcement raised alarms that it might serve to further demotivate and demoralize existing VHTs and create tension between CHEWs and VHTs. The selection criteria had an upper age limit of 35, which meant that there were many VHTs who would not be able to join this cadre, and the fact that CHEWs would be paid led to concerns about the willingness of VHTs to continue their work for free [8]. Ultimately, although the CHEW policy did gain initial approval in January 2019, it was recalled shortly thereafter due to “human resource gaps” [9]. Given the concerns raised about the CHEW policy, and the value VHTs provide their communities and country in promoting health and providing services, it is important to identify ways to better support VHTs and ameliorate their working conditions through the provision of appropriate and realistic incentive packages, which can help improve their performance and further encourage retention [1].

Globally, the recent Astana Declaration has reemphasized the role of PHC and contribution of CHWs in advancing universal health coverage [10]. As governments respond to this renewed wave of enthusiasm, it is necessary to address programmatic improvements in existing CHW programs. This study uses a discrete choice experiment (DCE) to elicit CHWs’ preferences for incentives in Uganda. DCEs quantitatively assess and identify how much an individual values certain attributes under consideration by making them go through a series of hypothetical alternatives to assert their choice; it also allows a better understanding of the trade-offs individuals are willing to make between attributes [11). DCEs have been widely used in health economics research, and more recently, among studies in low-and-middle-income countries to study incentive preferences of health workers [11-21]. In Uganda, a DCE was used with volunteer CHWs in family planning programs in 2011 to examine factors related to their motivation. The study identified recognition in form of t-shirts and badges, a mobile phone, and social prestige as some of the core elements associated with CHW motivation [7,15]. Given the ongoing debates on appropriate incentives for Uganda’s national cadre of CHWs, this study is conducted with a variety of CHWs including VHTs and CHPs, with a larger sample size, and across several geographic areas in Uganda to identify current incentives preferences amongst CHWs.

## METHODS

The DCE study was conducted in two phases. *Phase 1* was geared at identifying relevant and actionable incentive attributes or characteristics. *Phase 2* involved a quantitative DCE survey with the CHWs to collect choice data, presenting them with a series of incentive choices with varying attribute levels. Good research practice guidelines were followed in conducting and reporting the DCE [22].

### Phase 1: Identification of attributes and levels

The first step in designing a DCE is identification of the key service attributes that are important to the target group [11]. To do this, we conducted a series of focus group discussions (FGDs) and in-depth interviews (IDIs) with CHWs, CHW supervisors, national- and policy-level stakeholders, and non-governmental organizations (NGOs) in Uganda’s Lira, Mayuge, and Wakiso Districts in May 2019. The supervisory and policy-level stakeholders included Health Assistants, District Health Educators, Health-in-Charges, as well as representatives from Uganda’s MoH, Makerere University’s School of Public Health, BRAC, AMREF, and Living Goods. *Phase 1* had a total of 114 participants. Ten FGDs were conducted across the three districts (31 respondents in Lira, 29 in Mayuge, and 30 in Wakiso). Twenty-four IDIs, with five respondents in Lira, seven in Mayuge, five in Wakiso, and seven with national stakeholders were conducted. Participants were purposively selected, in collaboration with the district-level leadership. FGDs and IDIs were held by trained facilitators; FGD and IDI duration ranged from one to two hours and were held in the relevant local language(s), audio-recorded, and translated and transcribed into English.

The FGDs utilized a nominal group technique (NGT) to elicit and rank incentive attributes. The facilitator asked participants to think about the aspects of their jobs that are the most important to them. The attributes were listed on a white-board as they were shared one-by-one with the group until no new attributes were identified. Tally marks were placed against attributes based on the number of times they were stated to then identify 6-8 most important attributes. Definitions and understanding of the top-ranked attributes was discussed and clarified. Then, through discussion with the participants, attribute levels (eg, tiers of payment, or types of transportation) were identified. The interviews produced a list of seven attributes. This list of attributes and levels was reviewed with several national-level stakeholders for validation of whether these seemed relevant and practical from a policy and pragmatic standpoint. This process resulted in the list of attributes and levels presented in **Table 1**.

**Table 1:** Final set of attributes and levels

Attribute	Levels	Definition
Stipend	50 000 USH (US$13.50)/month	Refers to the desired amount of monetary compensation provided to CHWs on a monthly basis.
	100 000 USH (US$27.00)/month	
	150 000 USH (US$40.50)/month	
Refresher trainings	Quarterly	Refers to regularity and consistency of trainings that occur after the initial training provided to CHWs to support their knowledge and skills-building.
	Bi-annual	
	Annual	
	Every two years	
Identification	Identity badges	Identification is ways in which CHWs can be identified by the community and health care facilities.
	Branded uniforms (eg, T-shirts)	
	Branded protective gear (eg, umbrella)	
Availability of tools	Paper job-aids/manuals only	Refers to tools that CHWs can use to support their ability to work.
	Work mobile phones with payment plan	
Means of transport	Bicycle	Refers to ways in which CHWs can travel to and from required locations (eg, communities, health care facilities, training venues)
	Motorcycle	
	Transportation allowance	
Recognition	Membership in VHT club	Refers to ways in which to best recognize CHWs for their contributions and work.
	Low-interest credit for starting business	
	Priority health care for immediate family	
Workload	4 hours a day / 2 days a week	Refers to the amount of work that CHWs have.
	8 hours a day / 2 days a week	
	4 hours a day / 4 days a week	

VHT – village health team, CHW – community health worker

### Experimental design and construction of choice sets

The choice sets were unlabeled, consisting of two incentive alternatives and an opt-out labelled “neither” (**Table 2**). Full profiles were used, and respondents would be prompted to select the alternative they preferred. To minimize the number of choice sets to a number that can be pragmatically administered in a survey and reduce the cognitive burden on the respondent, we used a fractional factorial experimental design to generate 12 choice tasks [7]. A main effects orthogonal design was used where each attribute was statistically independent of each other and balanced (each level of the attribute occurred equally) using Sawtooth Software [23]. A dominant alternative was manually added to act as a rationality test to make it 13 choice sets [11].

**Table 2:** Sample choice set presented to the community health workers in Phase 2 of the study

Attributes	Job A	Job B	Neither
**Stipend**	50 000 USH (US$13.50)/months	100 000 USH (US$27.00)/month	
**Identification**	Branded uniforms (eg, T-shirts, aprons, caps)	Identity badges	
**Refresher Training**	Refresher training every 2 years	Refresher training 2 times a year	
**Availability of Tools**	Paper job-aids/manuals	Mobile phones with airtime for work only	
**Means of Transport**	Transport allowance	No transport	
**Recognition**	Career progression (eg, training certificates)	Membership of a CHW club/association	
**Workload**	4 hours a day for 2 days a week	8 hours a day for 2 days a week	
**Which alternative do you choose?**	YES OR NO	YES OR NO	NEITHER

CHW – community health worker

### Phase 2: DCE survey

**Figure 1:**
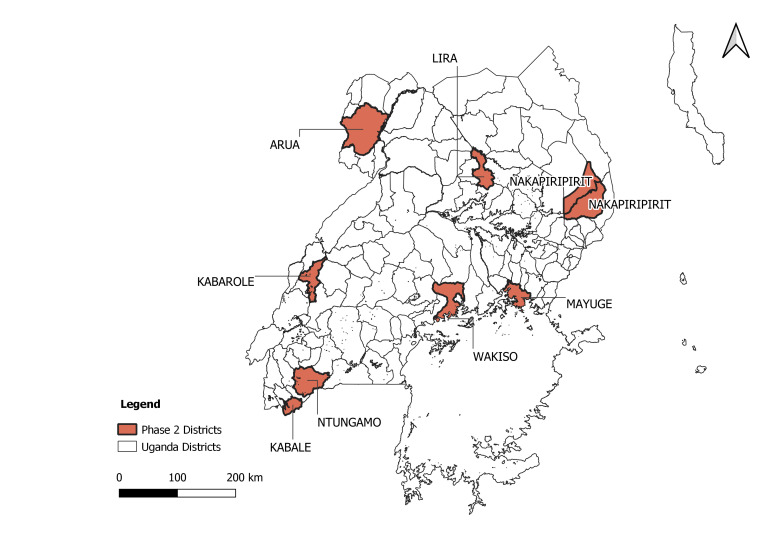
Districts where Phase 2 of the study was conducted.

To ensure that the *Phase 2* results captured preferences of CHWs working across a range of geographic settings and working conditions, eight districts were selected for fielding the *Phase 2* questionnaire: Lira, Mayuge, Wakiso, Ntungamo, Kabale, Arua, Kabarole, and Nakapiripirit (Figure 1). These include peri-urban areas, mountainous hard-to-reach districts, pastoral areas with predominantly nomadic communities, areas with refugee and displaced populations, and areas with predominantly traditional communities. The *Phase 2* survey questionnaire included questions about respondent demographics, content and years of training as a CHW, socioeconomic status, and current level and quality of supervision, and 13 choice sets [24]. The questionnaire was pretested and piloted with a subset of 20 respondents and changes made to the content and wording of the questions to account for any conceptual overlap and lack of clarity. The questionnaire was translated into eight languages and administered by a field team member fluent in the language, or along with a local leader fluent in the language. The questionnaire was implemented on Androids using ODK data collection software to 399 CHWs [24,25]. Sample size estimation followed rule of thumb by Johnson and Orme [26] and can be found in the published study protocol [27]. **Table 2** presents an example of a choice set presented to the CHWs.

### Statistical analysis

χ^2^ test was used to compare the categories for the descriptive statistics. Discrete choice models are based on the random utility theory (RUT). The marginal utilities were estimated using a conditional logit model as it is consistent with RUT [28]. Conditional logit models estimate choices probabilities as a function of the attributes of the alternatives under consideration [28]. The utility function of the main effects conditional logit model was specified as follows:

U_njt_ = β_0_ + β_1_ × Stipend_njt_ + β_2_ × Training_njt_+ β_3_ × Identification_badges_njt_ + β_4_ × Identification_uniforms_njt_ + β_5_ × Identification_protective_gear_njt_ + β_6_ × Tools_njt_ + β_7_ × Transport_bicycle_njt_ + β_8_ × Transport_allowance_njt_ + β_9_ × Transport_mortorcycle_njt_ + β_10_ × Recognition_credit_njt_ + β_11_ × Recognition_health care_njt_ + β_12_ × Recognition_progression_njt_ + β_13_ × workloadnjt+ε_njt_,

where *U_njt_* was the utility a CHW *n* derived from selecting incentive alternative *j* in choice task *t*, *β_0_* was the alternative specific constant for the opt-out, *β_1_* to *β_13_* were parameters to be estimated, *ε_njt_* were error terms which were assumed to be independently and identically distributed following type-1 extreme value distribution. Stipend was a continuous variable and modelled linearly, training and workload were modelled linearly based on a preference for more regular training and lower workloads, and the remaining variables were dummy coded variables for the levels of identification, tools of trade, transport and recognition attributes.

The conditional logit model however assumes homogeneity of preferences and relies on the property of independence of irrelevant alternatives (IIA) holding, which might not be the case [29]. A mixed multinomial logit (MMNL) was estimated as they relax restrictions due to IIA that are required for conditional logit models and permit modelling of repeated choices by the same individual, as in the case of this study [30]. The MMNL model is an extension of the standard conditional logit model that allows for attribute coefficients to be randomly distributed with a specified probability distribution such as normal, uniform, lognormal [30]. The MMNL utility function was similar to the conditional logit utility function above. All parameters were specified to be random and normally distributed except Stipend which was restricted to a lognormal distribution. We specified 500 Halton draws. The MMNL model resulted in means which represented preferences and standard deviations which captured preference heterogeneity.

The MMNL model means were used to calculate the relative importance CHWs placed on the incentive attributes. This was accomplished by taking the absolute value of the estimated means of the parameters of each attribute [31]. This was multiplied by the difference between the highest and lowest values of the levels of the attributes. The resultant value was the maximum effect which we took it proportion in relation to the total for each attribute to compute the relative importance estimates [31].We computed the willingness to accept estimates by taking the ratio between the negative coefficient of the non-monetary attributes and the monetary one since all the parameters in the conditional logit model were fixed [32]. The monetary attribute was monthly stipend and represented the amount of money the CHWs were willing to accept in Ugandan shillings. The delta method was used to estimate the confidence intervals.

Data analysis was conducted using Clogit, WTP, and Mixlogit commands on Stata 15.1 [33,34].

### Ethical approval

The research protocol was approved by the Population Council’s Institutional Review Board (PC IRB 872) and Makerere University College of Health Sciences’ Institutional Review Board (Protocol 657).

## RESULTS

A total of 399 CHWs were interviewed in Phase 2 of the study. **Table 3** presents the demographic characteristics of the CHW respondents. The average age of the CHWs was 44.5 years, they were 59% female, and about 70% had a secondary education or higher. The respondents comprised of 58% VHT only (government recognized cadre of workers), 14% community health promoters (CHP- supported by various NGOs), 11 percent of the respondents played a VHT supervisor role and 17 percent CHWs who were both VHTs and CHPs.

**Table 3:** Demographic characteristics of CHWs (n = 399) in a discrete choice experiment

Respondent characteristics	N = 399	%
**Age, mean years (SD)**	44.5 (10.6)	
**Sex (%)**		
Male	164	41
Female	235	59
**Education (%):**		
Never attended	6	1.5
Nursery	0	0
Primary	114	29
Secondary	242	61
College/University/Vocational	36	9
**Religion (%):**		
Christian	363	91
Muslim	34	8.5
Other	2	0.5
**Marital Status (%):**		
Never married	12	3
Married / Living together	324	81
Divorced / Separated	28	7
Widowed	35	8.8
**Type of CHW (%):**		
VHT only	231	58
CHP (NGO-supported)	55	14
VHT Coordinator	44	11
Both VHT/CHP	66	17
Other	3	0.8
**Avg. years worked as a VHT/CHP, mean (SD)**	10 (5.1)	

CHW – community health worker, NGO – non-governmental organization, VHT – village health teams, CHP – community health promoter, SD – standard deviation

To understand the context further, the surveys assessed current practices and environments that support CHWs (**Table 4**). Around 86% of CHWs walk to work, with the remaining 14% using a combination of bicycles, motorcycles, vehicles, and public transportation. About 30% of CHWs travel greater than an hour and 15% travel for more than 2 hours to get to the farthest households in their catchment area. The majority of CHW respondents (95.5%) indicated that they have received monetary compensation for their work. Sixty-three percent of those who received compensation reported primarily receiving it ad hoc (eg, during events); 22% reported receiving it quarterly. The types of non-monetary incentives received included trainings, health services, supplies for work, among others. Training is often considered an ‘incentive’ due to the transportation/travel refunds provided or the opportunities for advancement that are linked with it.

**Table 4:** Current practices and supportive environment for the community health workers

	N	%
**Work-related transportation (%):**		
Walk	344	86.2
Bicycle	33	8.3
Public transportation	11	2.8
Motorbike	11	2.8
**Travel time to farthest household (%):**		
<15 min	28	7
≥15 min &<30 min	61	15.3
≥30 min &<1 h	128	32.1
≥1 h &<2 h	122	30.6
≥2 h	60	15
**Primary income source (non-VHT/CHP-related) (%):**		
Agriculture	198	60.9
Business owner	92	28.3
Other*	35	10.8
**Financial compensation for VHT/CHP work (%):**		
Yes	381	95.5
No	18	4.5
**Type of compensation received (%):**		
Financial	45	11.8
Non-Financial^†^	8	2.1
Both	328	86.1
**Payment timing (%):**		
Weekly	1	0.3
Monthly	54	14.5
Quarterly	82	22
Ad hoc (when there is an event)	235	63
Other	1	0.3

VHT – village health team, CHW – community health worker

*Includes teacher, other health professionals, community/religious leaders.

†Includes additional training, food, clothes and other goods, supplies for work.

**Table 5** presents results of the conditional logit (Model 1) and the MMNL models (Model 2). The MMNL model provided a better fit for the data. Overall, CHW preferred incentives with higher salaries (β = 0.10, 95% CI = 0.06, 0.15) while the opt-out was less preferred. Reliable transportation and identification were significantly preferred (Table 5). The CHWs had a strong preference for identity badges (β = 1.61, 95% CI = 0.72, 2.49), branded uniforms (β = 1.04, 95% CI = 0.45, 1.63) and protective branded gear (β = 0.76, 95% CI = 0.32, 1.21) compared to no identification. In the transport attribute, the strongest preference was for a bicycle (β = 1.86, 95% CI = 1.06, 2.67), followed by motorcycle (β = 1.81, 95% CI = 1.27, 2.34) and transport allowance (β = 1.37, 95% CI = 0.65, 2.10).

**Table 5:** CHW incentive preferences using conditional logit and a mixed multinomial logit models

	Model 1a (*)			Model 2b (†)					
**Attribute**	**Mean**	**SE**	**95% CI**	**Mean**‡,	**SE**	**95% CI**	**SD**§	**SE**	**95% CI**
**Stipend**	0.04	0.03	(-0.01, 0.09)	0.10‖	0.02	(0.06, 0.15)	0.11‖	0.02	(0.60, 1.13)
**Refresher trainings** (Ref: quarterly training)	-0.35¶	0.16	(-0.01, -0.03)	-0.53‖	0.18	(-0.89, -0.17)	0.25‖	0.05	(0.15, 0.35)
**Identification:**									
No identification	*Reference*			*Reference*					
Identity badges	0.86¶	0.43	(-0.66, -0.03)	1.61‖	0.45	(0.72, 2.49)	-0.12	0.37	(-0.84, 0.61)
Branded uniforms	0.35	0.33	(0.01, 1.71)	1.04‖	0.30	(0.45, 1.63)	0.36¶	0.17	(0.03, 0.69)
Branded protective gear	0.51‖	0.19	(-0.30, 0.99)	0.76‖	0.23	(0.32, 1.21)	-0.35¶	0.15	(-0.64, -0.05)
**Availability of tools:**									
Paper job aids and manuals	*Reference*			*Reference*					
Work mobile phones	0.24‖	0.05	(0.14, 0.34)	0.41‖	0.08	(0.25, 0.56)	0.51‖	0.09	(0.34, 0.68)
**Means of transport:**									
No transport	*Reference*			*Reference*					
Bicycle	1.35‖	0.33	(0.70, 2.01)	1.86‖	0.41	(1.06, 2.67)	-0.39	0.21	(-0.80, 0.02)
Transport allowance	1.29‖	0.36	(0.58, 2.00)	1.37‖	0.37	(0.65, 2.10)	0.55‖	0.18	(0.19, 0.91)
Motorcycle	1.54‖	0.28	(0.99, 2.09)	1.81‖	0.27	(1.27, 2.34)	1.32‖	0.17	(0.99, 1.66)
**Recognition:**									
Being a member of VHT club	*Reference*			*Reference*					
Low interest credit for starting small business	-0.02	0.26	(-0.52, 0.49)	0.36	0.24	(-0.11, 0.83)	0.70‖	0.14	(0.43, 0.97)
Priority health care for immediate family members and referrals	0.02	0.18	(-0.33, 0.37)	0.32¶	0.16	(0.01, 0.62)	0.84‖	0.14	(0.58, 1.11)
Career progression	0.38	0.46	(-0.52, 1.27)	0.92‖	0.45	(0.04, 1.79)	0.35¶	0.16	(0.03, 0.67)
**Workload** (Ref: minimum workload)	-0.18	0.21	(-0.59, 0.23)	-0.5‖	0.20	(-0.89, -0.12)	0.12	0.28	(-0.43, 0.67)
**Opt-out (alternative specific constant)**	-4.38‖	0.44	(-5.23, -3.53)	-4.73	0.67	(-6.05, -3.41)	0.27	0.76	(-1.21, 1.75)
No. of respondents	399			399					
No. of observations	14364			14364					
Log-likelihood	-2966.03			-2868.75					
Likelihood ratio (χ^2^)	928.75			1097.57					

CI – confidence interval, SD – standard deviation, SE – standard error, VHT – village health team, CHW – community health worker

*Conditional logit model.

†Mixed logit model using Stata's mixlogit command and 500 Halton draws.

‡The coefficient (mean) represents the mean marginal utility of each attribute conditional on other attributes in a choice set.

§The standard deviation of the random coefficients represents the inter-respondent heterogeneity in preferences.

¶*P* < 0.05.

‖*P* < 0.01.

There was a preference for more frequent training opportunities. Higher workloads were less preferred (β = -0.5, 95% CI = -0.89, -0.12). The use of mobile phones as tools of trade were preferred (β = 0.41, 95% CI = 0.25, 0.56) compared to paper job-aids, and there was a preference for recognition in the form of priority health care (β = 0.32, 95% CI = 0.01, 0.62) and career progression (β = 0.92, 95% CI = 0.04, 1.79) over membership in a CHW club. Overall, there was preference heterogeneity across most attributes according to the values of the standard deviations.

**Figure 2:**
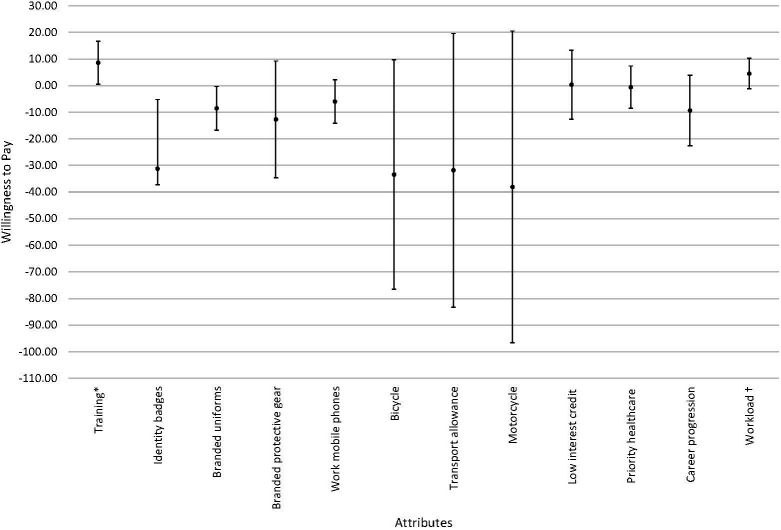
Graphical representation of willingness to accept estimates. *Ref: Quarterly training. †Ref: 4 hours/day, 2 days/week.

The relative importance estimates (Appendix S1 in the **Online Supplementary Document**) suggested that transport was the most important attribute, followed by identification, refresher training, salary, workload, recognition, and availability of tools. In the willingness to accept analyses (**Figure 2**), CHWs were willing to accept an increase in salary/stipend of USH 85 600 (US$23) for a one unit decrease in training frequency if everything else was kept constant. Furthermore, they were willing to accept a USH 31 240 (US$8.5) decrease in salary/stipend for identity badges, and a decrease of USH 85 300 (US$23) for branded uniforms to no identification if everything else was kept constant.

## DISCUSSION

The results of this study highlight findings that have been identified in other CHW programs globally, and validates that CHWs are not solely motivated by monetary compensation alone [5,6,13,15,20,21,35-40]. This study also further identifies that while CHWs need a reasonable salary or monetary compensation for their role, other aspects of the job are also important to consider [5,6,20,35,37]. In this study, we identified that CHWs in Uganda valued jobs with reliable transportation, appropriate identification in the form or identity badges or branded uniforms, consistent training, higher salaries, less intense workload, recognition in the form of priority health care for themselves and community members they refer to health care facilities, opportunities for career progression, and mobile job-aids, in this order. We also identified that the CHWs in Uganda were willing to accept a decrease in salary in exchange for more frequent training, as well as for formal identification such as identify badges and branded uniforms.

Transportation was identified as a key motivator, with the provision of bicycles being strongly preferred, closely followed by motorcycles and transportation allowances. Providing means of transport enables CHWs better access to communities, and enables them to perform their responsibilities more efficiently and effectively [5,15,21,36,37]. CHWs also reported having to pay out-of-pocket for transportation to attend meetings or reach communities, which affects their own livelihoods especially when it is not reimbursable (15,37,40]. Out of the three, transportation allowances were the least preferred, likely because there may be concerns with ensuring that the allowances reach CHWs in a consistent and timely manner; this is also consistent with the previous DCE conducted in Uganda [5,15,20,37]. It is also critical to consider the longevity and sustainability of these transport options (eg, bicycle, motorcycle) as they are not a one-time provision, and likely require maintenance.

Provision of refresher trainings are strongly desired by CHWs [15,37,39-42]. Trainings allow CHWs to not only reinforce old skills and learnings, but also learn new ones – which enables them to better support their communities; this could be through learning how to better identify and treat illnesses, or learning new methods to engage the community [15,41]. In this study, there was a strong preference for quarterly-held refresher trainings, as compared to the less frequently held ones. While refresher trainings were considered to be an incentive within this context, it should be noted that trainings are technically not incentives, given that it is through trainings that CHWs can function and provide services within their roles [36]. Trainings should be considered a guaranteed fixture in their job, whereas features of trainings can be considered incentives. Trainings can signify potential career progression, supporting CHWs in finding more meaning and value in their work [15,40]. Additionally, trainings are often associated with monetary rewards (eg, honorariums and transportation allowances), which can also be considered forms of financial incentives [37].

Means of identification is critical for CHWs, as it simultaneously asserts their credibility and promotes their recognition by communities and health care facilities [35,40,41]. This study indicated that CHWs preferred having a method of identification over no identification at all, strongly preferring the option for identification cards. They also indicated preferences for branded uniforms and branded protective gear. Ways in which to recognize CHWs included career progression, priority health care for immediate family members, low interest credit for starting a small business, and membership in a VHT club. CHWs preferred career progress and priority health care, akin to other studies [7,42]. Given that CHWs find value in the work that they do, this could mean that CHWs want to see improvements in their working conditions and the types of tangible recognition options available to them [15].

Job-aids generally can help provide CHWs with more credibility and ability to do their jobs [38,41]. Mobile phones were preferred over the use of paper-based job aids. Mobile phones can encompass a wide range of activities that can support CHWs’ responsibilities, ranging from having job-aids built-in, being a data collection device, and supporting communication with both the community and supervisors [36]. CHWs also preferred a lighter workload (4 hours a day for 2 days a week) compared to heavier ones (8 hours a day for 2 days a week; 4 hours a day for 4 days a week). Heavy workloads play a significant role in demotivating CHWs, and result in poor job satisfaction [37]. This is often exacerbated by the lack of financial compensation or incentives provided to CHWs, as a higher workload can also result in less availability for income-generating activities [13].

Several discrete choice experiments conducted in other African countries have comparable results to our study. In Uganda, a DCE was conducted amongst 183 CHWs, identifying incentive packages that comprised of a branded T-shirt, badge, and bicycle, followed by a mobile phone as a priority [15]. In a DCE study conducted among 199 CHWs in Kenya, it was noted that CHWs had a preference for community appreciation for their work [21]. In another DCE study in Ghana involving community health officers, opportunities for career development was identified as a priority [43]. A DCE study with 66 community-based mobilizers in Tanzania suggested that identity cards, bimonthly trainings, supervision, and a monthly flat renumeration were critical, similar to findings from our study [35]. The importance of non-monetary incentives (eg, recognition, opportunities for advancement) has also been identified by the 2018 WHO guideline on health policy and system support to optimize CHW programs. This study contributes to the evidence on the types of incentive packages and bundling of financial and non-financial incentives that would be effective in improving the performance of CHW programs [1].

DCEs help provide a more concrete understanding of incentive preferences considered desirable by CHWs. By conducting FGDs and IDIs with CHWs, CHW supervisors, and policy-/national-level stakeholders in *Phase 1*, our study enabled a more robust understanding of stakeholder perspectives, which consequently helped inform pragmatic list of incentive attributes that were tested as part of the DCE. While this study is not nationally representative, it provides results that are more broadly generalizable across different types of CHWs, given the broad geographic reach of the study, and the enrollment of different types of CHWs (eg, VHTs, CHPs) in the study group. However, as with most DCEs, a significant limitation is that the job/incentive attributes presented to CHWs were hypothetical and constitute stated preferences. CHW preferences may also change based on their prior experiences and organizational affiliations. It is possible that the hypothetical scenarios were not fully understood, or that the CHWs chose options that they felt were more likely to be implemented. To better understand how the scenarios and preferences indicated in this study affect and influence CHW motivation and retention, it would be beneficial to practically test them. An appropriate next step would be to test, under effectiveness settings, how implementation of a combination of incentives identified by this study can pragmatically yield improvements in CHW performance and motivation, as well as community health outcomes.

## CONCLUSION

The study reinforces the fact that salary, though an important attribute, alone does not address the needs of CHWs in Uganda. The study provides important insights on the wide range of job characteristics that are identified as critical from the CHW perspective. CHW programs, though effective, continue to face challenges in terms of retention and poor performance, in part due to a working environment that is not supportive. It is important to recognize factors in CHW work environments that limit their work satisfaction, motivation, and ultimately their performance. By working towards improving their working conditions, it can ideally lead to improvements not only in their retention, but also for community health outcomes.

## Additional material

Online Supplementary Document
