# Supplementary material for: Understanding community health worker incentive preferences in Uganda using a discrete choice experiment
Source: J Glob Health. 2021 Mar 10;11:07005. doi: 10.7189/jogh.11.07005 (PMC7956012; doi:10.7189/jogh.11.07005)
Supplement: Online Supplementary Document [file jogh-11-07005-s001.pdf]

Appendix S1: Relative importance estimates

Attribute	Absolute Value (Effect)	Difference in Levels	Maximum Effect	Relative Importance Estimates	Ranking
Stipend	0.1	15	1.5	0.17	4
Refresher Trainings	0.53	3	1.59	0.18	3
Identification	1.61	1	1.61	0.18	2
Availability of Tools	0.41	1	0.41	0.05	7
Means of Transport	1.86	1	1.86	0.21	1
Recognition	0.92	1	0.92	0.10	6
Workload	0.5	2	1	0.11	5
**Total**			8.89		
